# A functional polymorphism in the promoter of *TUG1* is associated with an increased risk of ischaemic stroke

**DOI:** 10.1111/jcmm.14499

**Published:** 2019-07-02

**Authors:** Ye‐Sheng Wei, Jun Yang, Yong‐Ling He, Xiang Shi, Zhi‐Neng Zeng

**Affiliations:** ^1^ Department of Laboratory Medicine Affiliated Hospital of Guilin Medical University Guilin China

**Keywords:** ischaemic stroke, luciferase activity, polymorphism, taurine‐upregulated gene 1, transcriptional factor

## Abstract

Taurine‐upregulated gene 1 (*TUG1*), a kind of long non‐coding RNAs (lncRNAs), was up‐regulated in ischaemic stroke (IS) with the function of promoting neuron apoptosis. In this study, we aimed to investigate the association of *TUG1* polymorphisms with IS risk. The *TUG1* polymorphisms were genotyped using a custom‐by‐design 48‐Plex SNPscan kit. The promoter activity was measured using the dual luciferase reporter assay. Relative expression of *TUG1* in IS patients was analysed using quantitative PCR and the binding of *TUG1* rs2240183 polymorphism to transcription factor was analysed using chromatin immunoprecipitation (ChIP) assay. The rs2240183 CT/CC genotypes and C allele in the promoter of *TUG1* were associated with an increased risk of IS (CT/CC vs. TT: adjusted OR = 1.70, 95% CI, 1.16‐2.49, *P* = 0.006; C vs. T: adjusted OR = 1.47, 95% CI, 1.12‐1.93, *P* = 0.005). Logistic regression analysis showed that the rs2240183 was a risk factor of IS besides TC, TG, HDL‐C, LDL‐C, VLDL‐C, Apo‐A1, Apo‐B and NEFA. Further functional analysis revealed that the *TUG1* rs2240183 C allele exhibited higher transcriptional activity and *TUG1* expression levels (*P* < 0.01). The ChIP assay showed that the rs2240183 C allele binds to transcriptional factor GATA‐1. These findings indicate that the rs2240183 C allele was associated with a higher risk of IS possibly by binding to GATA‐1 and elevating *TUG1* levels.

## 1 INTRODUCTION

Atherosclerosis is a disease characterized by the progressive accumulation of lipids and plaque formation in the arteries. Initially, there are no symptoms. When the blood flow is obstructed by atherosclerotic plaque, it will result in severe diseases, such as stroke and coronary artery disease.^1^, ^2^ Stroke is a major cause of death and disability worldwide. In China, there are about 2.5 million new cases and 1.5 million deaths each year.^3^ Ischaemic stroke (IS), the most common type, accounted for about 73%‐87% of all strokes.^4^, ^5^, ^6^ Risk factors of IS included smoking, hypertension, diabetes, obesity as well as genetic factors.^7^, ^8^, ^9^, ^10^, ^11^ For example, siblings or mothers of stroke case patients had 2‐4 times higher risk of stroke compared to controls.^10^ Our previous work identified some susceptibility loci of IS, such as rs9722 AA in S100 calcium‐binding protein B, rs1804826 T in growth differentiation factor‐15 and rs4705342 TT in the promoter of miR‐143/145.^12^, ^13^, ^14^

In the human genome, at least 98% regions are non‐coding transcripts, which have been defined as transcriptional ‘noise’ for a long time. According to the nucleotide (nt) sequence length, non‐coding RNAs can be divided into two major categories: small (<200 nt) and long non‐coding RNAs (≥200 nt, lncRNAs).^15^, ^16^, ^17^ Recently, the expression, function and mechanisms of lncRNAs had been widely studied in various diseases, including IS. For example, taurine‐upregulated gene 1 (*TUG1*) was found to be up‐regulated in cerebral ischaemic animals and oxygen‐glucose deprived (OGD) cells, with the function of promoting neuron apoptosis by sponging microRNA (miR)‐9 and increasing the expression of pro‐apoptosis gene Bcl‐2‐like 11 (Bcl2l11).^15^, ^18^ These findings highlighted that *TUG1* may be involved in the pathological process of IS.

Previous work has reported that single nucleotide polymorphisms (SNPs) related to lncRNAs may affect individual's susceptibility to IS.^19^, ^20^, ^21^, ^22^ The risk variants included rs217727 TT genotype in lncRNA H19, rs145204276 del/del genotype in the promoter of lncRNA growth arrest‐specific 5 (GAS5) and rs1537378 GG, rs2184061 AA, rs7044859 AA and rs7865618AA in the antisense non‐coding RNA in the INK4 locus (ANRIL).^19^, ^20^, ^21^, ^22^ To date, no study has reported the association between the *TUG1* polymorphisms and risk of IS. In this case‐control study, we investigated whether the *TUG1* polymorphisms were associated with the occurrence of IS in a Chinese Han population.

## 2 MATERIALS AND METHODS

### 2.1 Ethics, consent and permissions

The study protocol was approved by the Ethics Committee of the Affiliated Hospital of Youjiang Medical University for Nationalities. All patients agreed to participate in the study and provided informed consent.

### 2.2 Consent to publish

The participants signed the consent to publish the data.

### 2.3 Study population

The study population consisted of 571 controls and 556 patients with IS. The patients were consecutively enrolled from the Affiliated Hospital of Youjiang Medical University for Nationalities, Guangxi, China, between October 2010 and September 2015. Detailed information of sample collection has been described in our previous work.^14^, ^23^ Briefly, the diagnosis of IS was determined according to clinical symptoms, physical examinations and cranial magnetic resonance imaging and/or cranial computed tomography. We excluded those patients with haemorrhagic, autoimmune or chronic inflammatory diseases and family history of stroke. Controls were healthy volunteers after physical examination in the hospital. We excluded those patients who had brain tumours, autoimmune diseases, haematological disorder and family history of stroke. The following clinical data were collected: age, sex, smoking status, total cholesterol (TC), triglyceride (TG), high‐density lipoprotein cholesterol (HDL‐C), low‐density lipoprotein cholesterol (LDL‐C), very low‐density lipoprotein cholesterol (VLDL‐C), apolipoprotein A1 (Apo‐A1), apolipoprotein B (Apo‐B), homocysteic acid (Hcy) and non‐esterified fatty acid (NEFA). The interval time between IS onset and biochemical test was within 1 day. All patients were unrelated Han Chinese living in Guangxi province.

### 2.4 SNPs selection

We selected SNPs using the following criteria: (a) tagSNPs in lncRNA *TUG1*; (b) in silico prediction revealed potentially functional SNPs in the promoter region of *TUG1*; (c) minor allele frequency is more than 5% in Chinese Han population. Finally, five SNPs (ie, rs2240183, rs5749201, rs5753409, rs7284767 and rs8139350) were selected for further analysis.

### 2.5 Genotyping

About 2‐3 mL of ethylene diamine tetraacetic acid‐anticoagulated peripheral blood was taken from each participant before treatment. Genomic DNA was extracted using a salting‐out method.^24^ The SNPs were genotyped using a custom‐by‐design 48‐Plex SNPscan kit on an ABI3730XL sequencer (Genesky Biotechnologies Inc, Shanghai, China).^25^ This technique was based on double ligation and multiplex fluorescence PCR. For quality control, about 5% of all samples were randomly selected for Sanger sequencing and the results were 100% consistent.

### 2.6 Plasmid construction and dual‐luciferase reporter assay

*TUG1* promoter sequence containing the rs2240183 CC or rs2240183 TT genotype was amplified using the following primers: 5’‐TCCTTATCCCAAAGGCTTCC‐3’ (forward) and 5’‐ATGCCAGAGCAGGAGAAAGA‐3’ (reverse). The PCR products were inserted into a pGL3 basic vector (Promega, Madison, WI, USA). The constructed plasmids were verified using Sanger sequencing. Human embryonic kidney cell line 293 (HEK293) was cultured in medium supplemented with 10% foetal bovine serum. The cells were plated into 24‐well plates and transfected with 1 μg of rs2240183 C or rs2240183 T together with 50 ng of internal control (Renilla luciferase vector pRL‐SV40). Luciferase activity was measured at 48 hours after transfection using the Dual Luciferase Reporter Assay Kit (Promega).

### 2.7 Quantitative PCR (qPCR)

Total RNA was isolated from peripheral blood cells of 81 IS patients using a commercial kit (Qiagen, Hilden, Germany) following the manufacturer's manual. After the reverse transcription reaction, quantitative PCR was performed using SYBR Master Mix on an ABI 7900HT real‐time PCR machine (Applied Biosystems, CA, USA). The primer sequences of *TUG1* were described previously 26: 5’‐TTCCTACCACCTTACTACTGACG‐3’ (forward) and 5’‐GGAGGTAAAGGCCACATC‐3’ (reverse). *β‐actin* was used as an internal control and primer sequences were as follows: 5’‐ TTGCCGACAGGATGCAGAA‐3’ (forward) and 5’‐GCCGATCCACACGGAGTACT‐3’ (reverse). Relative expression of *TUG1* was determined using the ‐ΔCt method.

### 2.8 Chromatin immunoprecipitation (ChIP) assay

ChIP assay was performed using a commercial kit (ThermoFisher Scientific, Waltham, MA, USA) following the manufacturer's protocol. Briefly, HEK293 cells were cross‐linked in 1% formaldehyde and sonicated to produce fragments of 200‐1000 bp. Antibodies against GATA‐1 and rabbit IgG (Abcam) were used to treat the samples to introduce immunoprecipitation. After incubation overnight at 4°C, the immune complexes were washed, reversely cross‐linked and finally re‐suspended in 20 μL of double–distilled H_2_O. The diluted DNA and input DNA were amplified by PCR using the primers: 5’‐TCCTTATCCCAAAGGCTTCC‐3’ (forward) and 5’‐TGTTCCAGCTTCACCAAAGA‐3’ (reverse). The PCR products were analysed using Sanger sequencing.

### 2.9 Statistical analysis

Statistical analysis was performed using SPSS version 19.0 software (SPSS, Chicago, IL, USA). Chi‐square test was used to evaluate whether the study population deviates from Hardy‐Weinberg equilibrium (HWE). Odds ratios (ORs), 95% confidence intervals (CIs) plus associated *p* values were computed using χ^2^ test. ORs were adjusted according to age, sex, TC, TG, HDL‐C, LDL‐C, VLDL‐C, Apo‐A1, Apo‐B, Hcy and NEFA. For multiple testing, Bonferroni corrected *p* value was set as 0.0125. Linkage disequilibrium (LD) and haplotype analysis were carried out using SHEsis software (http://analysis.bio-x.cn/myAnalysis.php).^27^ Logistic regression was used to identify risk factors of IS *P* values less than 0.05 were considered statistically significant.

## 3 RESULTS

### 3.1 Characteristics of the study population

The characteristics of the study population are presented in Table 1. The distributions of age, gender, smoking status and TC level were not significantly different between cases and controls. The levels of TG, LDL‐C, VLDL‐C and Apo‐B were higher whereas the levels of HDL‐C, Apo‐A1, Hcy and NEFA were lower in IS patients (*P* < 0.05).

**Table 1:** Baseline characteristics of the study population

Variables	Controls, n = 571	Patients with IS, n = 556	*P* value
Age, y (mean ± SD)	59.2 (±11.2)	60.2 (±10.9)	0.13
Male/Female	379/192	393/163	0.12
Smoking, yes/no	175/395	192/364	0.17
TC, mmol/L	4.83 ± 0.89	4.78 ± 1.02	0.40
TG, mmol/L	1.36 ± 1.02	1.88 ± 1.43	<0.001
HDL‐C, mmol/L	1.51 ± 0.40	1.29 ± 0.41	<0.001
LDL‐C, mmol/L	2.32 ± 1.00	2.83 ± 0.98	<0.001
VLDL‐C, mmol/L	0.71 ± 0.51	0.84 ± 0.66	<0.001
Apo‐A1, g/L	1.76 ± 1.16	1.23 ± 0.26	<0.001
Apo‐B, g/L	0.76 ± 0.30	1.00 ± 0.30	<0.001
Hcy, μmol/L	14.85 ± 3.69	14.21 ± 5.82	0.03
NEFA, mmol/L	0.71 ± 0.30	0.55 ± 0.28	<0.001

Apo‐A1, apolipoprotein A1; Apo‐B, apolipoprotein B; Hcy, homocysteic acid; HDL‐C, high‐density lipoprotein cholesterol; IS, ischaemic stroke; LDL‐C, low‐density lipoprotein cholesterol; NEFA, non‐esterified fatty acid; SD, standard deviation; TC, total cholesterol; TG: triglyceride; VLDL‐C, very low‐density lipoprotein cholesterol.

### 3.2 Main effect of *TUG1* polymorphisms on IS risk

The genotype and allelic frequencies of the five SNPs between cases and controls are summarized in Table 2. The genotype distributions in controls conformed to HWE. The rs2240183 CC and CT/CC genotypes were associated with an increased risk of IS with an adjusted OR of 2.26 and 1.70 respectively (CC vs TT: 95% CI, 1.22‐4.19; *P* = 0.009; CT/CC vs TT: 95% CI, 1.16‐2.49; *P* = 0.006). Similarly, increased risk of IS was also observed in allele comparison with adjusted OR of 1.47 (95% CI, 1.12‐1.93; *P* = 0.005). No significant association between rs5749201, rs5753409, rs7284767 and rs8139350 and IS risk was found.

**Table 2:** Association between* TUG1* polymorphisms and risk of IS

Polymorphisms	Controls, n = 571 (%)	IS, n = 556 (%)	Adjusted OR (95% CI)^a^	*P* value
rs2240183				
TT	245 (42.9)	179 (32.2)	1.00	
CT	271 (47.5)	280 (50.4)	1.61 (1.08‐2.41)	0.02
CC	55 (9.6)	97 (17.4)	2.26 (1.22‐4.19)	0.009
CT/CC	326 (57.1)	377 (67.8)	1.70 (1.16‐2.49)	0.006
T	761 (66.6)	638 (57.4)	1.00	
C	381 (33.4)	474 (42.6)	1.47 (1.12‐1.93)	0.005
rs5749201				
TT	284 (49.7)	252 (45.3)	1.00	
AT	234 (41.0)	254 (45.7)	1.03 (0.70‐1.52)	0.90
AA	53 (9.3)	50 (9.0)	0.95 (0.50‐1.80)	0.87
AT/AA	287 (50.3)	304 (54.7)	0.99 (0.69‐1.43)	0.96
T	802 (70.2)	758 (68.2)	1.00	
A	340 (29.8)	354 (31.8)	0.97 (0.73‐1.29)	0.85
rs5753409				
GG	461 (80.7)	431 (77.5)	1.00	
AG	105 (18.4)	117 (21.0)	1.04 (0.65‐1.66)	0.86
AA	5 (0.9)	8 (1.4)	0.62 (0.11‐3.44)	0.58
AG/AA	110 (19.3)	125 (22.5)	1.01 (0.64‐1.60)	0.96
G	1027 (89.9)	979 (88.0)	1.00	
A	115 (10.1)	133 (12.0)	0.98 (0.64‐1.49)	0.92
rs7284767				
AA	311 (54.5)	331 (59.5)	1.00	
AG	220 (38.5)	190 (34.2)	0.75 (0.50‐1.11)	0.15
GG	40 (7.0)	35 (6.3)	0.74 (0.35‐1.55)	0.42
AG/GG	260 (45.5)	225 (40.5)	0.75 (0.51‐1.09)	0.13
A	842 (73.7)	852 (76.6)	1.00	
G	300 (26.3)	260 (23.4)	0.80 (0.59‐1.08)	0.14
rs8139350				
CC	470 (82.3)	458 (82.4)	1.00	
CG	95 (16.6)	90 (16.2)	0.83 (0.51‐1.35)	0.46
GG	6 (1.1)	8 (1.4)	0.65 (0.13‐3.27)	0.60
CG/GG	101 (17.7)	98 (17.6)	1.21 (0.76‐1.94)	0.43
C	1035 (90.6)	1006 (90.5)	1.00	
G	107 (9.4)	106 (9.5)	0.84 (0.54‐1.29)	0.42

CI, confidence interval; IS, ischaemic stroke; OR, odds ratio; TUG1, taurine‐upregulated gene 1.

a Adjusted by age, sex, total cholesterol, triglyceride, high‐density lipoprotein cholesterol, low‐density lipoprotein cholesterol, very low‐density lipoprotein cholesterol, apolipoprotein A1, apolipoprotein B, homocysteic acid and non‐esterified fatty acid.

LD analysis showed that the rs5749201, rs5753409, rs7284767 and rs8139350 were in moderate LD and haplotype analysis was then performed. As shown in Table 3, eight common haplotypes were detected. The AGGC haplotype tended but did not reach the significance to decrease the risk of IS compared to the TGAC haplotype (OR = 0.78, 95% CI, 0.61‐1.00, *P* = 0.05).

**Table 3:** Haplotype analysis of *TUG1* polymorphisms with IS risk

rs5749201	rs5753409	rs7284767	rs8139350	Controls (%)	IS (%)	OR (95% CI)	*P* value
T	G	A	C	515 (45.1)	495 (44.5)	1.00	
A	G	G	C	189 (16.5)	142 (12.8)	0.78 (0.61‐1.00)	0.05
A	G	A	C	134 (11.7)	163 (14.7)	1.27 (0.98‐1.64)	0.08
T	A	A	C	91 (8.0)	89 (8.0)	1.02 (0.74‐1.40)	0.91
T	G	G	C	92 (8.1)	80 (7.2)	0.91 (0.65‐1.25)	0.55
T	G	A	G	81 (7.1)	67 (6.0)	0.86 (0.61‐1.22)	0.40
A	G	A	G	15 (1.3)	14 (1.3)	0.97 (0.46‐2.03)	0.94
T	A	G	C	14 (1.2)	14 (1.3)	1.04 (0.49‐2.21)	0.92

CI, confidence interval; IS, ischaemic stroke; OR, odds ratio; TUG1, taurine‐upregulated gene 1.

Only frequency greater than 1% is presented.

### 3.3 Stratification analysis

Stratification analysis was performed between the *TUG1* polymorphisms and clinical characteristics of IS. As shown in Table 4, patients carrying rs5749201 AT/AA genotypes had lower levels of TC, HDL‐C and Apo‐A1 compared to the rs5749201 TT carriers (*P* = 0.03, 0.003, and 0.007 respectively). However, no significant association was observed between rs2240183, rs5753409, rs7284767 and rs8139350 and clinical characteristics of IS.

**Table 4:** Stratification analysis of TUG1 polymorphisms and clinical characteristics of IS

Variables	rs2240183			rs5749201			rs5753409			rs7284767			rs8139350		
	TT	CT/CC	*P*	TT	AT/AA	*P*	GG	AG/AA	*P*	AA	AG/GG	*P*	CC	CG/GG	*P*
TC, mmol/L	4.80 ± 1.05	4.78 ± 1.00	0.78	4.88 ± 0.92	4.70 ± 1.08	0.03	4.81 ± 1.03	4.69 ± 0.97	0.22	4.79 ± 1.01	4.77 ± 1.03	0.82	4.78 ± 1.01	4.80 ± 1.03	0.84
TG, mmol/L	1.94 ± 1.39	1.86 ± 1.44	0.54	1.86 ± 1.26	1.89 ± 1.55	0.81	1.85 ± 1.28	1.97 ± 1.83	0.41	1.94 ± 1.62	1.79 ± 1.07	0.22	1.89 ± 1.48	1.83 ± 1.16	0.71
HDL‐C, mmol/L	1.27 ± 0.41	1.31 ± 0.40	0.31	1.35 ± 0.42	1.25 ± 0.39	0.003	1.30 ± 0.41	1.27 ± 0.39	0.48	1.29 ± 0.40	1.30 ± 0.41	0.70	1.28 ± 0.40	1.34 ± 0.44	0.18
LDL‐C, mmol/L	2.91 ± 0.97	2.79 ± 0.99	0.17	2.80 ± 1.04	2.86 ± 0.94	0.45	2.84 ± 0.98	2.80 ± 0.99	0.66	2.82 ± 1.02	2.85 ± 0.93	0.76	2.84 ± 0.97	2.79 ± 1.04	0.66
VLDL‐C,mmol/L	0.90 ± 0.78	0.80 ± 0.59	0.11	0.82 ± 0.65	0.85 ± 0.67	0.69	0.83 ± 0.62	0.84 ± 0.79	0.94	0.87 ± 0.80	0.79 ± 0.37	0.14	0.85 ± 0.70	0.76 ± 0.43	0.23
Apo‐A1, g/L	1.22 ± 0.24	1.23 ± 0.27	0.73	1.26 ± 0.26	1.20 ± 0.26	0.007	1.23 ± 0.26	1.21 ± 0.24	0.44	1.24 ± 0.26	1.21 ± 0.27	0.23	1.22 ± 0.26	1.25 ± 0.25	0.35
Apo‐B, g/L	1.00 ± 0.30	1.00 ± 0.30	0.97	1.02 ± 0.31	0.99 ± 0.29	0.20	1.00 ± 0.30	1.00 ± 0.31	0.86	1.01 ± 0.31	1.00 ± 0.29	0.83	1.00 ± 0.30	1.01 ± 0.28	0.80
Hcy, μmol/L	14.08 ± 5.04	14.28 ± 6.16	0.70	14.58 ± 6.14	13.91 ± 5.53	0.17	14.33 ± 6.14	13.81 ± 4.55	0.38	14.08 ± 5.84	14.41 ± 5.79	0.50	14.27 ± 6.03	13.96 ± 4.72	0.63
NEFA, mmol/L	0.57 ± 0.29	0.54 ± 0.28	0.14	0.55 ± 0.28	0.55 ± 0.29	0.90	0.55 ± 0.29	0.53 ± 0.28	0.40	0.54 ± 0.28	0.55 ± 0.28	0.61	0.55 ± 0.27	0.52 ± 0.32	0.31

Apo‐A1, apolipoprotein A1; Apo‐B, apolipoprotein B; Hcy, homocysteic acid; HDL‐C, high‐density lipoprotein cholesterol; IS, ischaemic stroke; LDL‐C, low‐density lipoprotein cholesterol; NEFA, non‐esterified fatty acid; TC, total cholesterol; TG, triglyceride; TUG1, taurine‐upregulated gene 1; VLDL‐C, very low‐density lipoprotein cholesterol.

### 3.4 Multivariate logistic regression analysis

Logistic regression was carried out to determine the risk factors of IS. As shown in Table 5, the risk factors included TC (OR = 1.73; 95%CI, 1.29‐2.31), TG (OR = 1.72; 95%CI, 1.43‐2.07), HDL‐C (OR = 0.15; 95%CI, 0.08‐0.31), LDL‐C (OR = 1.74; 95%CI, 1.43‐2.13), Apo‐A1 (OR = 0.01; 95%CI, 0.01‐0.02), Apo‐B (OR = 226.07; 95%CI, 80.70‐633.36), NEFA (OR = 0.16; 95%CI, 0.09‐0.31) and rs2240183 (OR = 1.56; 95%CI, 1.30‐1.87) (*p* all < 0.001).

**Table 5:** Logistic regression analysis for identifying risk factors of IS

Variables	B	OR (95% CI)	*P* value
TC	0.55	1.73 (1.29‐2.31)	<0.001
TG	0.54	1.72 (1.43‐2.07)	<0.001
HDL‐C	‐0.53	0.15 (0.08‐0.31)	<0.001
LDL‐C	0.56	1.74 (1.43‐2.13)	<0.001
Apo‐A1	‐4.62	0.01 (0.01‐0.02)	<0.001
Apo‐B	5.42	226.07 (80.70‐633.36)	<0.001
NEFA	‐1.81	0.16 (0.09‐0.31)	<0.001
rs2240183	0.45	1.56 (1.30‐1.87)	<0.001

Apo‐A1, apolipoprotein A1; Apo‐B, apolipoprotein B; CI, confidence interval; HDL‐C, high‐density lipoprotein cholesterol; IS, ischaemic stroke; LDL‐C, low‐density lipoprotein cholesterol; NEFA, non‐esterified fatty acid; OR, odds ratio; TC, total cholesterol; TG, triglyceride.

### 3.5 **The** rs2240183 **C allele increased the transcriptional activity**

**Figure 1:**
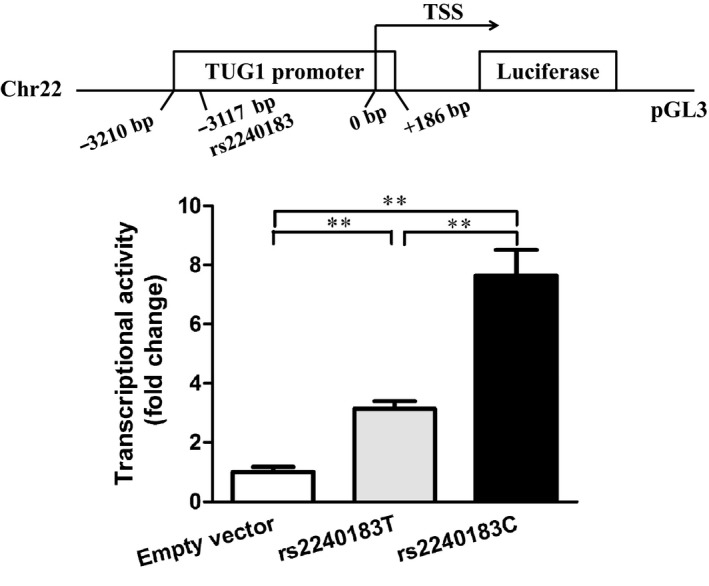
The rs2240183 C allele increased the transcriptional activity. A, Schematic representation of the plasmid construction of *TUG1* promoter. A 3396 bp promoter sequence of *TUG1* containing the rs2240183 C or T allele (from −3210 to +186bp) was cloned into a pGL3 basic vector. TSS, transcriptional start site. B, The plasmids were transfected into HEK293 cells and luciferase activity was measured 48 h after transfection (**P < 0.01)

To determine whether the rs2240183 in the promoter of *TUG1* affected the luciferase activity, we constructed plasmids containing the rs2240183 C or T allele and measured the reporter activity. The schematic representation of the plasmid construction is shown in Figure 1A. As shown in Figure 1B, both rs2240183 C and T allele exhibited a higher luciferase activity compared to the empty vector (**P < 0.01). Importantly, the rs2240183 C allele had a higher luciferase activity compared to the rs2240183 T allele (**P < 0.01).

### 3.6 **The** rs2240183 **CC genotype was associated with increased levels of *TUG1***

**Figure 2:**
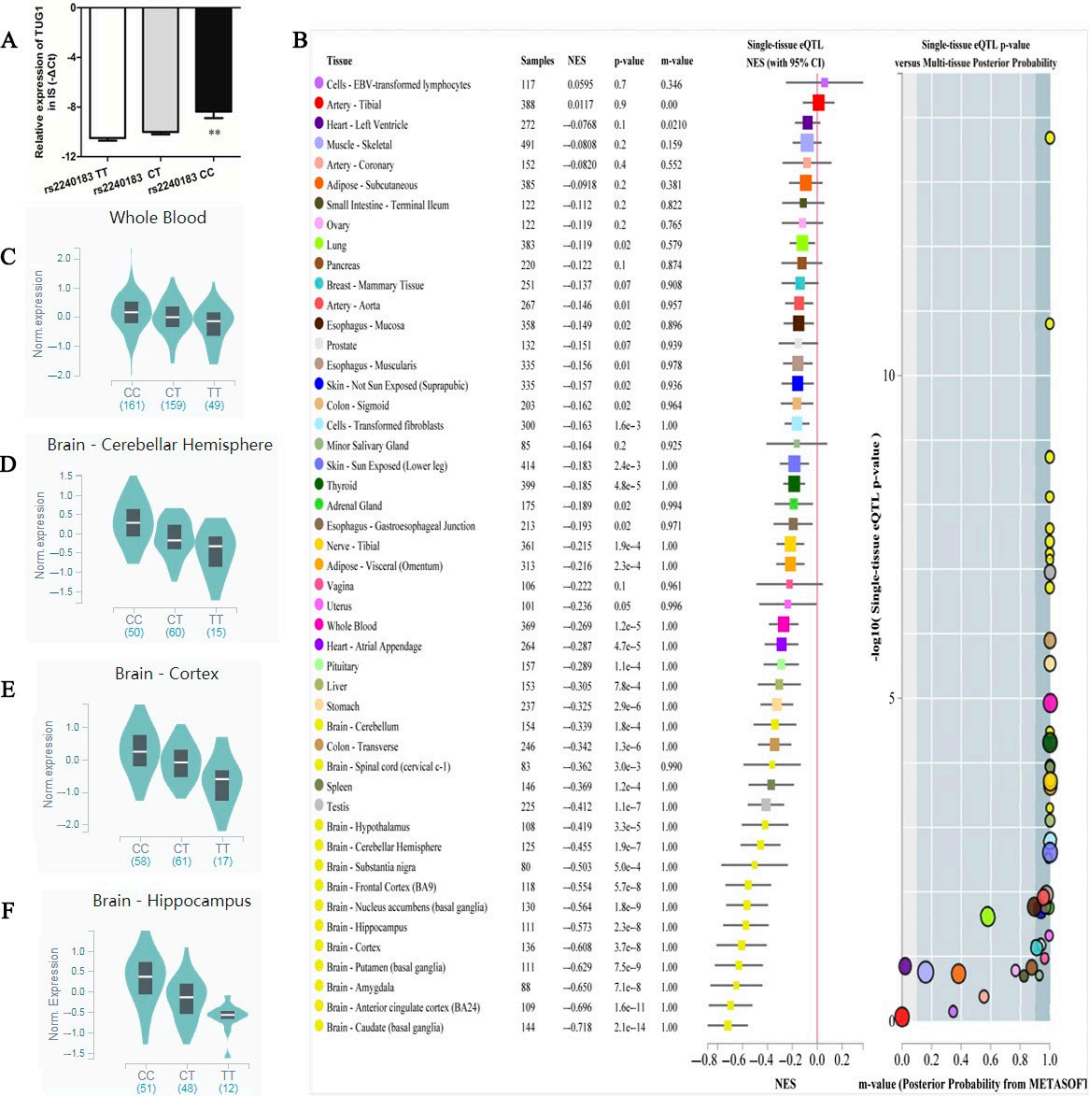
The rs2240183 CC genotype was associated with increased levels of *TUG1*. A, quantitative PCR was used to examine the expression levels of *TUG1* in ischaemic stroke patients. Compared to rs2240183 TT carriers, rs2240183 CC carriers had increased levels of *TUG1* (**P < 0.01)*.* Expression Quantitative Trait Loci analysis of rs2240183 with gene expression in single tissue (B), whole blood (C), cerebellar hemisphere (D), cortex (E) and hippocampus (F)

To determine whether the rs2240183 influenced *TUG1* expression, we analysed the levels of *TUG1* in 81 IS patients using qPCR. As shown in Figure 2A, the rs2240183 CC carriers had increased levels of *TUG1* compared to the rs2240183 TT carriers (**P < 0.01)*.* These findings were consistent with results from the expression Quantitative Trait Loci (eQTL) (Figure 2B). The rs2240183 CC genotype was associated with higher gene expression in several single tissues, such as whole blood, cerebellar hemisphere, cortex and hippocampus (Figure 2C‐F) (*P* < 0.001).

### 3.7 **The** rs2240183 **C allele binds to transcription factor GATA‐1**

**Figure 3:**
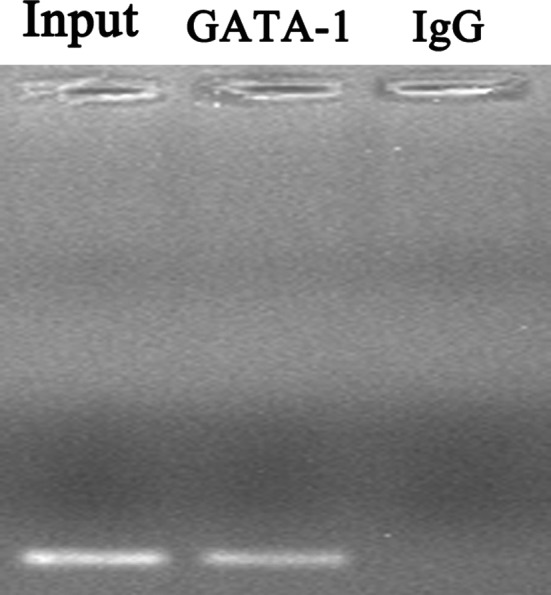
GATA‐1 binds to the promoter region of *TUG1* containing the rs2240183 CC genotype. ChIP was performed using anti‐GATA‐1 antibody and anti‐IgG antibody in HEK293 cells. The promoter region of *TUG1* containing the rs2240183 was analysed using PCR‐ electrophoresis

In silico analysis predicted that rs2240183 C but not rs2240183 T binds to transcription factor GATA‐1. ChIP assay was then used to validate the allele‐specific transcription factor binding. PCR‐electrophoresis revealed that the DNA fragments immunoprecipitated specifically with the anti‐GATA‐1 antibody rather than non‐specific IgG (Figure 3). Further sequencing showed that the GATA‐1 binding region contained the rs2240183 CC genotype of *TUG1* promoter.

## 4 DISCUSSION

In this study, we presented two major findings. One is that *TUG1* related polymorphisms were associated with the aetiology of IS. Individuals carrying the rs2240183 CT/CC genotypes in the promoter of *TUG1* had a 1.70‐fold higher risk of IS and patients carrying the rs5749201 AT/AA genotypes had lower levels of TC, HDL‐C and Apo‐A1. Logistic regression revealed that the rs2240183 was a risk factor together with previously identified risk parameters such as TC, TG, HDL‐C, LDL‐C, Apo‐A1, Apo‐B and NEFA. The second major finding was that the rs2240183 C allele binds to transcription factor GATA‐1, increases the promoter activity and finally elevates the expression of *TUG1* at the transcriptional level. Taken together, these findings indicate that the rs2240183 CT/CC genotypes were associated with a higher risk of IS possibly by increasing *TUG1* levels.

*TUG1*, a 7.2 kb lncRNA, was initially discovered in taurine‐treated mouse retinal cells and played key roles in retinal development.^28^ It is also observed to be up‐regulated in nervous system diseases.^18^ In atherosclerosis, the up‐regulation of *TUG1* enhances endothelial cell apoptosis by regulating miR‐26a,^29^ promotes vascular smooth muscle cell proliferation by regulating miR‐21/PTEN axis 30 and stimulates proliferation and migration of endothelial cells via the Wnt pathway.^31^ Knockdown of *TUG1* ameliorates atherosclerosis by modulating fibroblast growth factor 1 via miR‐133a.^32^ Under ischemia, *TUG1* silencing promotes cell survival and decreases cell apoptosis by sponging miR‐9 and decreasing Bcl2l11 protein.^18^ All the above mentioned miRNAs are key mediators in the pathology of IS.^33^, ^34^, ^35^, ^36^ miR‐26a promotes angiogenesis in a rat model of IS via the PI3K/AKT and MAPK/ERK pathway.^33^ miR‐21 may be used a biomarker to differentiate between IS and transient ischaemic attack patients and overexpression of miR‐21 protects against ischaemic neuronal death.^34^, ^35^ miR‐9 mediates cell apoptosis by targeting Bcl2l11 in IS.^18^, ^36^ These findings suggest that *TUG1* may be an important regulator in the development of IS.

Previously, lncRNA related SNPs had been found to be associated with the risk of IS.^19^, ^20^, ^21^, ^22^ Zheng et al reported that carriers with lncRNA GAS5 rs145204276 del/del genotype had a 2.06‐fold higher risk of IS.^19^ Wang et al reported that carriers with lncRNA H19 rs217727 TT genotype had a 4.29‐fold increased risk of IS.^21^ The results were confirmed by Zhu and the colleagues.^20^ Additionally, four SNPs (ie, rs1537378, rs2184061, rs7044859 and rs7865618) in ANRIL were found to confer to the risk of atherothrombotic stroke.^22^ Based on this background, we suggested that SNPs in *TUG1* may be related to the risk of IS. Our results confirmed this hypothesis and we found that the rs2240183 CT/CC genotypes in the promoter of *TUG1* were associated with an increased risk of IS in Table 2. However, the rs2240183 was not associated with clinical characteristics of IS in stratification analysis as shown in Table 4. One possible reason may be that the reference is different. In Table 2, the reference is controls, whereas in Table 4, all the volunteers were IS patients and the reference is part of patients. Also we cannot exclude the possibility that the results may occur by chance. A similar phenomenon was also observed in overall analysis of rs5749201. Although no significant association of rs5749201 with IS risk was observed compared to controls (Table 2), rs5749201 was associated with TC, HDL‐C and Apo‐A in IS patients (Table 4). The reason for the discrepancy may be explained by the possibility mentioned above. rs2240183 being a risk factor was also identified using subsequent multivariate logistic regression analysis. Besides rs2240183, previously identified risk parameters including TC, TG, HDL‐C, LDL‐C, Apo‐A1, Apo‐B and NEFA were also detected in the regression model. These findings indicate that rs2240183 may be a biomarker for the aetiology of IS in the Chinese population.

Next, we explored the possible mechanism for rs2240183 C increasing the risk of IS. We firstly used in silico analysis to predict the binding of rs2240183 and transcriptional factor and found that rs2240183 C but not T allele can bind to transcription factor GATA‐1. In this study, CHIP assay was performed and the allele‐specific GATA‐1 binding was validated. These findings suggest that rs2240183 C promoted the expression of *TUG1* by binding to GATA‐1, which may be the possible mechanism for rs2240183 C increasing the risk of IS. It is well known that transcription factor binding to the promoter sequence may promote the transcriptional activity. We then evaluated whether rs2240183 influenced the transcriptional activity and *TUG1* expression using the dual‐luciferase reporter gene assay and qPCR technique. We found that the rs2240183 C allele exhibited a higher reporter activity and patients carrying the rs2240183 CC genotype had higher levels of *TUG1.* These findings were consistent with the results from eQTL in several tissues, such as whole blood, brain, spinal cord, spleen, testis, liver and stomach, further supporting that our findings were reasonable and reliable.

There are some limitations in this study. We collected hospital‐based controls and thus the selection bias cannot be removed completely. Because of lack of data of alcohol consumption, gene‐environment interaction analysis could not be performed. Because of these limitations, further investigations are necessary to confirm the significance of rs2240183 as a risk factor for IS. The interaction of *TUG1* polymorphisms with environmental factors is of great value.

In conclusion, we demonstrated that the rs2240183 CT/CC genotypes in the promoter of lncRNA *TUG1* were risk factors for the development of IS. We also observed that rs2240183 C was associated with higher promoter activity and *TUG1* expression levels. Furthermore, results from ChIP may provide an underlying mechanism for the observed association with the susceptibility of IS. Because IS is a disease with high mortality and disability rate, it is of great importance to understand the risk factors for the development and progression of IS, which will benefit for the early prevention and intervention of IS.

## CONFLICT OF INTERESTS

The authors confirm that there are no conflicts of interest.

## AUTHOR CONTRIBUTION

Ye‐Sheng Wei designed and wrote the manuscript. Jun Yang and Yong‐Ling He helped to perform experiments. Xiang Shi performed the statistical analysis. Zhi‐Neng Zeng prepared for figures. All authors reviewed the manuscript.
